# Characteristics of non-conveyed patients in emergency medical services (EMS): a one-year prospective descriptive and comparative study in a region of Sweden

**DOI:** 10.1186/s12873-020-00353-8

**Published:** 2020-08-10

**Authors:** Erik Höglund, Magnus Andersson-Hagiwara, Agneta Schröder, Margareta Möller, Emma Ohlsson-Nevo

**Affiliations:** 1 University Health Care Research Center, Faculty of Medicine and Health, Örebro University, Box 1613, 701 16 Örebro, Sweden; 2 Centre for Prehospital Research, Faculty of Caring Science, Work Life and Social Welfare, University of Borås, Borås, Sweden; 3 Department of Health Sciences in Gjøvik, Faculty of Medicine and Health Sciences, NTNU – Norwegian University of Science and Technology, Gjøvik, Norway; 4 Department of Surgery, Faculty of Medicine and Health, Örebro University, Örebro, Sweden

**Keywords:** Ambulance, Emergency medical services, Non-conveyance, Non-transport, Triage

## Abstract

**Background:**

There has been an increasing demand for emergency medical services (EMS), and a growing number of patients are not conveyed; i.e., they are referred to levels of care other than ambulance conveyance to the emergency department. Patient safety issues have been raised regarding the ability of EMS to decide not to convey patients. To improve non-conveyance guidelines, information is needed about patients who are not conveyed by EMS. Therefore, the purpose of this study was to describe and compare the proportion and characteristics of non-conveyed EMS patients, together with assignment data.

**Methods:**

A descriptive and comparative consecutive cohort design was undertaken. The decision of whether to convey patients was made by EMS according to a region-specific non-conveyance guideline. Non-conveyed patients’ medical record data were prospectively gathered from February 2016 to January 2017. Analyses was conducted using the chi-squared test, two-sample t test, proportion test and Mann-Whitneys U-test.

**Results:**

Out of the 23,250 patients served during the study period, 2691 (12%) were not conveyed. For non-conveyed adults, the most commonly used Emergency Signs and Symptoms (ESS) codes were unspecific symptoms/malaise, abdomen/flank/groin pain, and breathing difficulties. For non-conveyed children, the most common ESS codes were breathing difficulties and fever of unclear origin. Most of the non-conveyed patients had normal vital signs. Half of all patients with a designated non-conveyance level of care were referred to self-care. There were statistically significant differences between men and women.

**Conclusions:**

Fewer patients were non-conveyed in the studied region compared to national and international non-conveyance rates. The differences seen between men and women were not of clinical significance. Follow-up studies are needed to understand what effect patient outcome so that guidelines might improve.

## Background

Both nationally and internationally, the demand for emergency medical services (EMS) has been growing approximately 3–5% annually [1, 2], and 40–79% of assessed patients do not need EMS interventions [3–5]. The proportion of patients who are assessed but do not require EMS interventions has also reportedly increased [1]. Because of the increasing demand and the fact that growing numbers of EMS users do not need EMS interventions, an increasing number of patients are left at the scene of the incident without ambulance conveyance to the emergency department (ED) [3, 4].

The growing and changing demand has been attributed to the growing and ageing population [2, 6–8] and to the fact that younger patients, patients with socio-economic or educational disadvantages, and patients with no pre-existing health conditions contacting EMS at disproportionately high rates [1, 9, 10]. Internationally, non-conveyance rates have been reported to be between 29 and 42% [4, 7]; in Sweden specifically, published non-conveyance rates vary between 10 and 22% [10–12].

The decision not to convey a patient has been described by EMS clinicians as a complex process that involves a great deal of responsibility [11, 13, 14]. Informal decision-making processes are predominant [14], and non-conveyance is more common in remote areas than near population centres [11, 15]. Taking ambulance availability into account when choosing not to convey patients from remote areas might jeopardize patient safety, since studies have shown that patients living further away from the hospital present themselves to EMS with more severe conditions than patients living in urban areas close to the hospital [16–18].

When patients are not conveyed, they are assessed with guidelines based on vital signs and levels of urgency [13]. Vital signs have been shown to be poor indicators with which to assess the acute care needs of older patients presenting with unspecific complaints [19–21]. The importance of sex when evaluating vital signs is also unclear, and there are ambiguous results regarding differences in assessment and treatment between males and females for different types of conditions [16, 22]. Swedish legislation requires that health care providers perform continuous follow-up on the planning, execution, outcomes, and improvement of their services [23]. Despite these requirements, the guidelines and protocols in Sweden are heterogeneous, based on best practices and not validated for the EMS context [24]. Non-conveyance decisions are complex, and the corresponding guidelines have shown questionable accuracy in determining appropriate care levels for non-conveyed patients [14, 25–27].

To develop practices with higher level of patient safety, some studies have highlighted the need for further insights into the characteristics of the non-conveyed population [13, 16]. This study is part of a larger project called non-conveyance - go to other level of care (No-Go).

### Aim

The primary aim of this study was to describe and compare the proportion and characteristics of non-conveyed EMS patients, together with assignment data.

The secondary aim of this study was to describe if there were any differences between male and female sex.

## Methods

### Design

This study used a descriptive and comparative consecutive cohort design. The report follows the Strengthening the Reporting of Observational Studies in Epidemiology (STROBE) guidelines [28].

### Setting and context

The study was conducted in a region in central Sweden. The region contains three ambulance departments and three hospitals comprising one level I trauma centre and two smaller hospitals with limited intensive care unit (ICU) capacity. Twelve ambulances operate around the clock in the region, and an additional four ambulances are staffed during the daytime. These ambulances serve 295,000 inhabitants and receive approximately 30,000 assignments per year. The proportions of male and female inhabitants in the region were approximately equal in 2016, and the age-group distribution was as follows: 0–10 years, 12%; 11–17 years, 8%; 18–30 years, 17%; 31–45 years, 18%; 46–64 years, 23%; 65–80 years, 17%; and > 80 years, 5% [29].

In Sweden, registered nurses (RNs) have 3 years of education at the university level, and RN specialists have an additional year of higher education. Since 2005, Swedish regulations have required ambulances to be staffed with health care professionals who are authorized to prepare and administer drugs [30]. In practice, the law requires each EMS team in Sweden to include at least one RN who can administer drugs and be responsible for the provided care. In addition to the RN with or without a specialist education, an EMS team may include another RN, a specialist ambulance nurse, or an emergency medical technician (EMT) [31]. There is no national requirement to staff ambulances with RNs specializing in prehospital emergency care, although approximately 60% of all EMS clinicians in the studied region were specialized ambulance nurses. RNs and EMTs also worked within the regional EMS system. Among all EMS clinicians, 1/3 were women. During summertime, the number of substitute EMTs increases due to holidays and a shortage of RNs. In this study, EMS personnel in general are referred to as EMS clinicians.

In 2015, the studied region implemented non-conveyance guidelines that were restrictive in their design, enabling EMS clinicians to choose not to convey patients and, instead, to refer them to levels of care other than ambulance care during transport to the ED. The guidelines included a triage system, the Rapid Emergency Triage and Treatment System (RETTS), which contains both Emergency Signs and Symptoms (ESS) codes and vital signs [32, 33]. Additionally, the non-conveyance guidelines include exclusion criteria built on expert consensus, summarized in a checklist (Additional file 1 – Exclusion criteria for non-conveyance). RETTS was developed in Sweden for the ED and is not validated for prehospital assessments or non-conveyance decisions.

The triage system uses the patient’s main complaint, a description of the illness, and the signs and symptoms to assign the patient a specific ESS code and triage level. The studied region used a subset of ESS codes that did not include psychiatric disorders. The system triages patients into urgency levels and the time within which the patient should be assessed by a physician. The triage system has colour codes to indicate different urgency levels. Green and yellow are the lowest priority levels, meaning that the patient does not need immediate emergency care and can wait 3 h or more. The orange priority level means that the patient has urgent medical needs but can wait up to 20 min to be seen by a physician after the handover at the ED. Red indicates the highest priority level, meaning that the patient needs immediate care and assessment by a physician [32, 33].

**Fig. 1:**
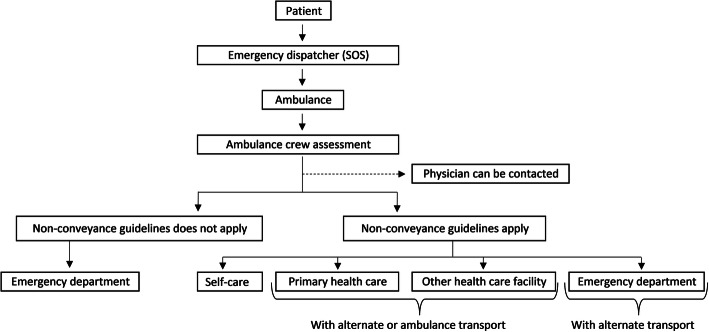
Flowchart for non-conveyance in the studied region Permission for reprint was obtained [11].

EMS clinicians have the option to consult a physician at the receiving hospital for advice when making a non-conveyance decision. EMS clinicians can arrange an appointment at a health care facility and arrange for public transport either to the ED or to another health care facility. When a patient is not conveyed, a non-conveyance document is created and given to the patient. The document contains information about the assessment of the patient, decisions regarding further health care contact, and where the patient should turn in case the condition worsens. A non-conveyed patient could be advised to provide self-care at home, to seek primary health care, or to go to the ED via personal or public transport (Fig. 1).

EMS clinicians perform a structured patient interview according to the Advanced Medical Life Support concept [34], assessing vital signs such as cognition Reaction Level Scale (RLS) [35], respiratory rate (breaths/min), blood oxygen saturation (SpO2), pulse (beats/min), blood pressure (mmHg), and temperature (degrees Celsius). For patients to be considered for non-conveyance, all vital signs should be in the normal range (Additional file 2 – Normal vital signs); additionally, children should have an ESS colour code of green, and adults should have a code of green or yellow. The patient or legal guardian must be able to communicate and understand the decision and information provided, and the patient must not need any drug administration, supervision or monitoring during transport to a health care facility (Additional file 1 – Exclusion criteria for non-conveyance).

### Sample and data collection

**Fig. 2:**
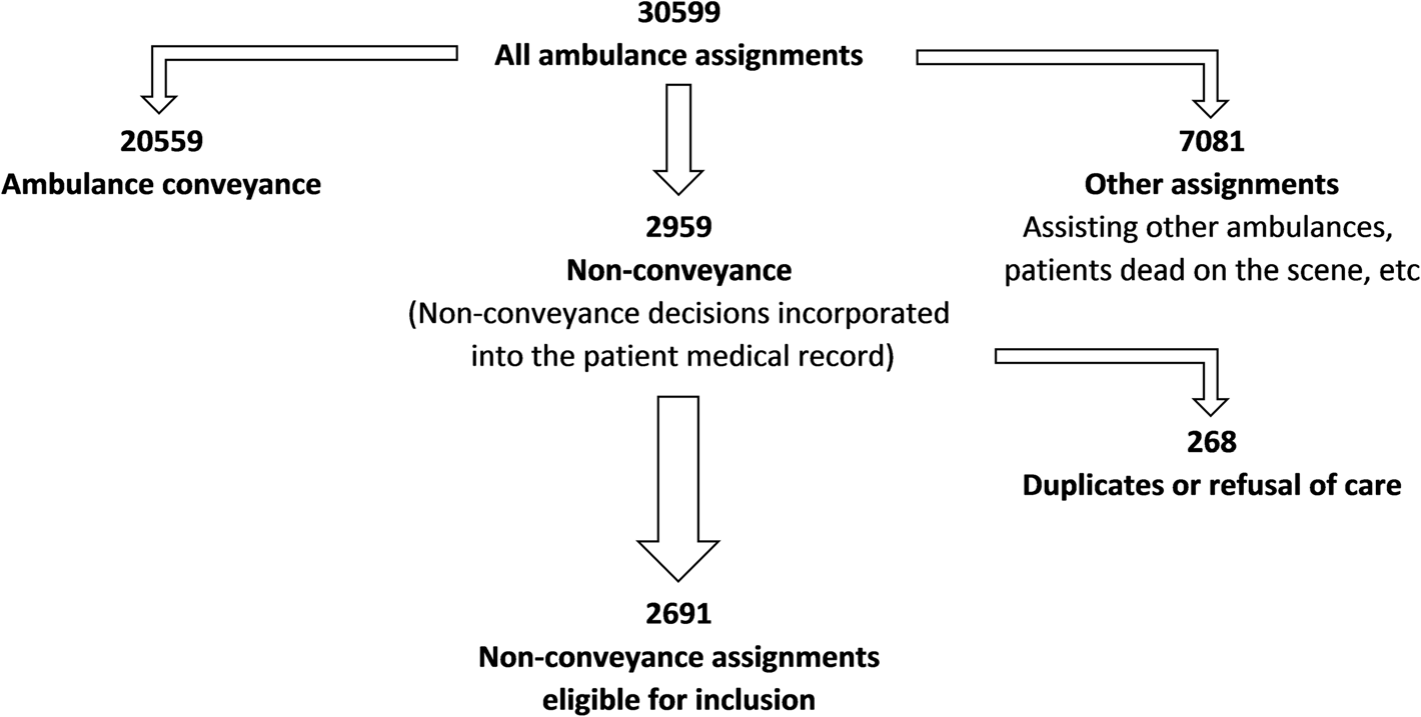
Flowchart of included ambulance assignments

All patients, including children and adults, who were visited by EMS from February 2016 to January 2017 but were not conveyed by the ambulance service were eligible for inclusion (Fig. 2). The data consisted of handwritten prehospital medical record data produced by EMS when patients were not conveyed. Data entry was structured a priori in SPSS version 25 with guidance and limitation instructions to ensure data quality. Data quality was checked both during and after entry. In total, 30% of all entered values were randomly selected and manually double-checked to verify that there were < 0.25% initial typographical errors. Typographical errors and deviant data were corrected.

We included all patients (children and adults) for whom the EMS clinician chose non-conveyance according to the guidelines.

We excluded all patients who refused care or ambulance conveyance as well as patients who were dead on the scene. See the inclusion and exclusion flowchart (Fig. 2).

### Data analysis

Demographic data were analysed using descriptive statistics. Categorical variables were described as numbers and percentages and continuous variables as both median, Q1-Q3 and mean, standard deviation (SD).

The data were tested for an approximately normal distribution by both graphical and numerical methods, including, box plots, histograms, and the Shapiro-Wilk test. Mann-Whitneys U-test and Two-sample t test was used for continuous data. The chi-squared and proportion test were used for categorical variables.

A *p*-value threshold of < 0.05 was used to test for statistical significance, and the Bonferroni correction was used for comparisons among > 5 groups.

The variables of respiratory rate, pulse, and temperature are dependent on age and were therefore reported as low, normal or high instead of as their actual values; see additional file 2. The last measured vital signs that formed the final triage was used in the analysis. Age groups were chosen to reflect on different stages of life and from non-conveyance guideline cut-offs between children and adults. Variables that were available for fewer than ten patients were not analysed. Data were analysed using STATA 15.1 data analysis software (College Station, Texas, USA: Stata Corp) and IBM SPSS Statistics for Windows, Version 24.0 (Armonk, NY: IBM Corp).

## Results

### Patient characteristics and ambulance utilization

In relation to the 23,250 patients served by the EMS, 2691 (12%) were not conveyed. The proportions of males and females were equal among the non-conveyed patients (*p* = 0.60). There was a statistically significant difference in non-conveyance rates across age groups (*p* < 0.001). There was a statistically significant difference for *Age* between non-conveyed male and female patients (*p* < 0.01). No statistically significant difference was found between males and females for *Age groups, Day of week* or *Time of day*. Most non-conveyance decisions were made in the evening, outside of office hours (Table 1).

**Table 1:** Patient characteristics and ambulance utilization, n (%) if not otherwise stated

	Non-conveyed patients, *n* = 2691	Male^a^, *n* = 1344	Female^a^, *n* = 1317
***Age****, Median (Q1-Q3)*	51 (25–73)	50 (25–72)	53 (26–77)
***Age groups***			
0–10 years	271 (10)	156 (12)	107 (8)
11–17 years	115 (4)	52 (4)	57 (4)
18–30 years	471 (18)	236 (18)	223 (17)
31–45 years	329 (12)	160 (12)	168 (13)
46–64 years	492 (18)	261 (19)	228 (17)
65–80 years	575 (21)	302 (22)	273 (21)
> 80 years	438 (16)	177 (13)	261 (20)
***Day of week***			
Monday	390 (15)	202 (15)	185 (14)
Tuesday	387 (14)	190 (14)	195 (15)
Wednesday	350 (13)	164 (12)	181 (14)
Thursday	351 (13)	173 (13)	174 (13)
Friday	375 (14)	191 (14)	180 (14)
Saturday	450 (17)	230 (17)	216 (16)
Sunday	388 (14)	194 (14)	186 (14)
***Time of day***^b^			
00:00–07:59	585 (22)	294 (22)	287 (22)
08:00–15:59	913 (34)	439 (33)	466 (35)
16:00–23:59	1167 (43)	594 (44)	555 (42)

*P*-value < 0.05 between non-conveyed male and female patients.

For > 5 groups, the Bonferroni correction was used, *p* < 0.007.

Mann-Whitneys U-test: *Age*. Proportion test: Sex.

Chi-squared test: *Age groups, Day of week* and *Time of day*.

^a^Sex missing 30 values, ^b^*Time of day* missing 26 values

**Fig. 3:**
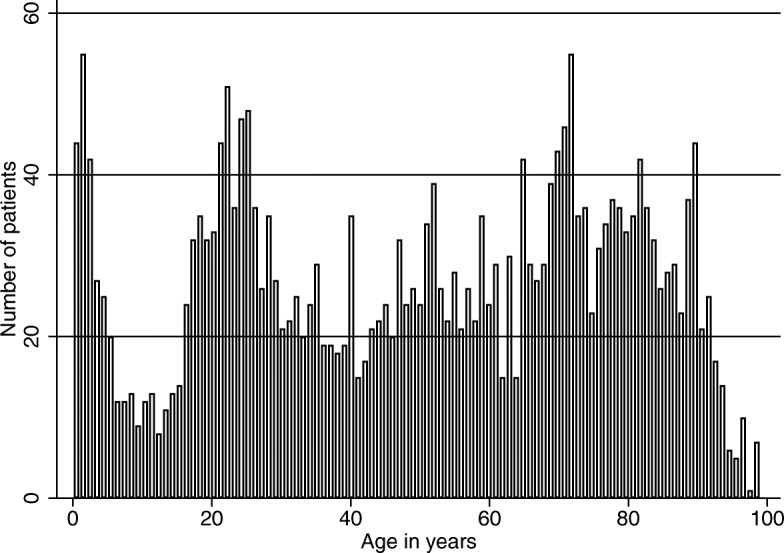
Frequency histogram showing the number of non-conveyance patients from February 2016 to January 2017

Patients were non-conveyed from all different ages between 0 and 99 years of age (Fig. 3).

### Vital signs of non-conveyed patients

For adult patients, vital signs were almost identical between males and females. There was a statistically significant difference (*P* < 0.01) for *temperature* and *blood oxygen saturation* between non-conveyed male and female adults. Patients’ vital signs were mostly within the normal range. Notably, although EMS clinicians are required to collect all data needed for a complete triage according to RETTS, between 6 and 19% of those data were incomplete. Between 1 and 14% of recorded data for non-conveyed patients were outside the guideline recommendations for non-conveyance. The vital signs that deviated most from normal were blood oxygen saturation (SpO_2_), with 14% of all recorded values between 90 and 95% (Table 2).

**Table 2:** Vital signs of non-conveyed adults (≥18 years), n (%) if not otherwise stated

	Non-conveyed patients, *n* = 2305	Male, *n* = 1136	Female, *n* = 1153
***Cognition***			
1	2085 (99)	1004 (99)	1067 (99)
≥ 2	16 (1)	9 (1)	7 (1)
Missing	(9)	(11)	(7)
***Respiratory rate****, Mean (SD)*	18 (3)	18 (3)	18 (3)
8–25	2083 (100)	1012 (100)	1052 (99)
> 30 or ≤ 7	1 (< 1)	0 (0)	1 (< 1)
26–30	9 (< 1)	3 (< 1)	5 (< 1)
*Missing*	*(9)*	*(11)*	*(8)*
***Blood oxygen saturation****, Mean (SD)*	98 (2)	97 (2)	98 (2)
96–100	1851 (86)	896 (85)	941 (86)
90–95	296 (14)	151 (14)	145 (13)
< 90	5 (< 1)	1 (< 1)	4 (< 1)
*Missing*	*(7)*	*(8)*	*63 (5)*
***Systolic blood pressure****, Mean (SD)*	140 (23)	139 (22)	140 (24)
90–199	2054 (98)	1004 (99)	1037 (98)
≥ 200	35 (2)	14 (1)	21 (2)
< 90	1 (< 1)	0 (0)	1 (< 1)
*Missing*	*(9)*	*(10)*	*(8)*
***Diastolic blood pressure****, Mean (SD)*	82 (12)	82 (13)	81 (13)
40–100	1784 (96)	866 (95)	905 (96)
> 100	83 (4)	47 (5)	36 (4)
< 40	0 (0)	0 (0)	0 (0)
*Missing*	*(19)*	*(20)*	*(18)*
***Pulse****, Mean (SD)*	86 (16)	85 (16)	87 (15)
50–120	2137 (99)	1042 (99)	1081 (99)
> 120	18 (1)	12 (1)	6 (1)
< 50	4 (< 1)	1 (< 1)	3 (< 1)
*Missing*	*(6)*	*(7)*	*(5)*
***Temperature****, Mean (SD)*	37 (1)	37 (1)	37 (1)
35–38.5	1959 (97)	943 (97)	1004 (98)
> 38.5	39 (2)	21 (2)	17 (2)
< 35.0	14 (1)	7 (1)	7 (1)
*Missing*	*(13)*	*(15)*	*125 (11)*

*P*-value < 0.05 between non-conveyed male and female adults.

Mann-Whitneys U-test: *Cognition*. T-test: All other vital signs.

Vital signs for children (Table 3) showed that the majority were within the normal range. *Pulse* and *respiratory rate* were the vital signs deviating most from normal, with one in five triaged patients outside the normal range for each of those variables (Additional file 2 – Normal vital signs). There was a statistically significant difference (*P* < 0.01) for *Diastolic blood pressure* between non-conveyed male and female children.

**Table 3:** Vital signs of non-conveyed children (0–17 years), n (%) if not otherwise stated

	Non-conveyed patients, *n* = 386	Male, *n* = 208	Female, *n* = 164
***Cognition***			
1	331 (100)	182 (100)	140 (100)
≥ 2	0 (0)	0 (0)	0 (0)
*Missing*	*(14)*	*(13)*	*(15)*
***Respiratory rate****, Mean (SD)*	25 (10)	25 (10)	25 (9)
Normal	267 (78)	147 (79)	112 (77)
High	73 (21)	37 (20)	33 (23)
Low	2 (1)	1 (1)	1 (1)
*Missing*	*(11)*	*(11)*	*(11)*
***Blood oxygen saturation****, Mean (SD)*	99 (1)	99 (1)	99 (1)
96–100	310 (97)	168 (97)	132 (96)
90–95	11 (3)	6 (3)	5 (4)
< 90	0 (0)	0 (0)	0 (0)
*Missing*	*(17)*	*(16)*	*(15)*
***Systolic blood pressure****, Mean (SD)*	121 (16)	121 (16)	120 (14)
90–199	108 (100)	50 (100)	54 (100)
*Missing*	*(72)*	*(76)*	*(67)*
***Diastolic blood pressure****, Mean (SD)*	75 (11)	71 (11)	78 (11)
50–120	89 (100)	42 (100)	43 (100)
*Missing*	*(77)*	*(80)*	*(74)*
***Pulse****, Mean (SD)*	114 (26)	114 (25)	114 (28)
Normal	251 (76)	143 (80)	101 (72)
High	74 (22)	34 (19)	38 (27)
Low	4 (1)	2 (1)	2 (1)
*Missing*	*(15)*	*(14)*	*(14)*
***Temperature****, Mean (SD)*	37 (1)	37 (1)	38 (1)
Normal	302 (100)	162 (100)	131 (100)
High	0 (0)	0 (0)	0 (0)
Low	1 (< 1)	1 (< 1)	0 (0)
*Missing*	*(22)*	*(22)*	*(20)*

*P*-value < 0.05 between non-conveyed male and female children.

Mann-Whitneys U-test: *Cognition*. T-test: All other vital signs.

### ESS coding and colours

One in five of the adult patients were assigned the ESS code for unspecific symptoms/malaise. Most (97%) had an ESS colour, which allowed them to be non-conveyed according to the non-conveyance guideline. ESS codes were represented in similar proportions for males and females (Table 4). No statistically significant difference was found between males and females for ESS codes or ESS colours *p* > 0.64.

**Table 4:** ESS codes and colours for non-conveyed adults (≥18 years), n (%) if not otherwise stated

	Non-conveyed patients, *n* = 2305	Male, *n* = 1136	Female, *n* = 1153
***ESS code***			
53 - Unspecific symptoms, malaise	345 (20)	169 (20)	175 (20)
6 - Abdomen, flank or groin pain	173 (10)	77 (9)	95 (11)
4 - Breathing difficulties	136 (8)	49 (6)	86 (10)
5 - Chest pain	99 (6)	46 (5)	52 (6)
11 - Vertigo, balance problems	97 (6)	40 (5)	57 (6)
50 - Hypoglycaemia	84 (5)	44 (5)	40 (5)
20 - Loss of consciousness	74 (4)	37 (4)	37 (4)
40 - Intoxication	62 (4)	38 (5)	24 (3)
47 - Fever, infection	59 (3)	26 (3)	31 (4)
14 - Back or neck pain	57 (3)	27 (3)	30 (3)
All other ESS-codes	56 (3)	29 (3)	27 (3)
30 - Injury, head/neck, strangulation, teeth	52 (3)	31 (4)	20 (2)
34 - Injury, legs/lower extremities	49 (3)	30 (4)	20 (2)
3 - Haemoptysis, epistaxis	45 (3)	24 (3)	21 (2)
19 - Headache, neuralgia	41 (2)	18 (2)	22 (3)
1 - Irregular heartbeat	39 (2)	19 (2)	20 (2)
9 - Seizures, epilepsy	38 2)	18 (3)	10 (1)
33 - Injury, shoulder/collarbone/arm/hand	38 (2)	22 (3)	15 (2)
15 - Extremity problems/pain	36 (2)	10 (1)	26 (3)
43 - Allergy	33 (2)	10 (1)	23 (3)
31 - Injury, abdomen/thorax/genitalia	29 (2)	21 (3)	< 10
12 - Neurological problems	27 (2)	12 (1)	15 (2)
16 - Urinary problems/pain	20 (1)	15 (2)	26 (3)
21 - Pregnancy and related problems	15 (1)	< 10	15 (2)
49 - Diabetes, high blood sugar	14 (1)	< 10	< 10
35 - Electrical/chemical accident	11 (1)	< 10	< 10
41 - Animal bites and toxic effects	11 (1)	< 10	< 10
*Missing*	*(24)*	*(26)*	*(22)*
***ESS colour***			
Green	1172 (72)	552 (72)	610 (71)
Yellow	416 (25)	198 (26)	218 (26)
Orange/Red	46 (3)	20 (2)	26 (3)
*Missing*	*(29)*	*(31)*	*(26)*

*P*-value < 0.05 between males and females. The Bonferroni correction was used, *p* < 0.002.

The chi-squared test was used to test statistical significance.

Table 5 shows that the ESS codes for almost one-third of the children were missing (missing document or not documented by the EMS clinicians); this category was more common than any single ESS code. Breathing difficulties and fever of unclear origin were the most commonly used ESS codes for children, representing 25% of all used ESS codes. No statistically significant difference was found between males and females for ESS codes or ESS colours *p* > 0.29.

**Table 5:** ESS codes and colours for non-conveyed children (0–17 years), n (%) if not otherwise stated

Children (0–17 years)	Non-conveyed patients, *n* = 386	Male, *n* = 208	Female, *n* = 164
***ESS code***			
104 - Breathing difficulties	41 (15)	28 (18)	12 (11)
154 - Fever of unclear origin	28 (10)	16 (10)	10 (9)
130 - Injury, head/neck, strangulation, teeth	24 (9)	16 (10)	8 (7)
109 - Seizures, epilepsy	20 (7)	11 (7)	9 (8)
143 - Allergy	15 (5)	11 (7)	3 (3)
106 - Abdomen, flank or groin pain	14 (5)	10 (6)	4 (4)
144 - Mouth blisters, sore throat, cold	14 (5)	9 (6)	5 (4)
146 - Foreign object in nose, airway, ear, internal tracts	13 (5)	5 (3)	8 (7)
153 - Unspecific symptoms, worried parents	12 (4)	5 (3)	7 (6)
147 - Dermal or dental infection or lump	10 (4)	6 (4)	4 (4)
*Missing*	*(29)*	*(26)*	*(31)*
***ESS colour***			
Green	194 (79)	102 (75)	87 (84)
Yellow	52 (21)	34 (25)	17 (16)
Orange/Red	0 (0)	0 (0)	0 (0)
*Missing*	*(36)*	*(35)*	*(37)*

*P*-value < 0.05 between males and females. The Bonferroni correction was used, *p* < 0.005.

The chi-squared test was used to test statistical significance.

In total, 1699 people had a designated non-conveyance level of care, of whom 329 were transported to the ED by some means other than an ambulance (19%), 496 were referred to primary health care (29%), and 874 were discharged for self-care (51%). For both children and adults, there were no significant differences between males and females (all *p* > 0.05) in the frequency of any non-conveyance destination. Children’s (0–17 years) non-conveyance destinations were similar to those of adults (Table 6).

**Table 6:** Non-conveyance destination, n (%) if not otherwise stated

Adults (≥18 years)	Non-conveyed patients, *n* = 2305	Male, *n* = 1136	Female, *n* = 1153
***Non-conveyance destination***			
Self-care	746 (51)	367 (52)	432 (51)
Primary health care	441 (30)	199 (28)	262 (31)
Emergency department	275 (19)	134 (19)	155 (18)
*Missing*	*(37)*	*(38)*	*304 (26)*
Children (0–17 years)	**Non-conveyed patients**, *n* = 386	**Male**, *n* = 208	**Female**, *n* = 164
***Non-conveyance destination***			
Self-care	128 (54)	67 (54)	58 (57)
Primary health care	55 (23)	25 (20)	25 (25)
Emergency department	54 (23)	33 (26)	18 (18)
*Missing*	*(39)*	*(40)*	*63 (38)*

*P*-value < 0.05 between males and females.

The chi-squared test was used to test statistical significance.

Emergency department and primary health care is a non-conveyance destination via personal or public transport.

## Discussion

The primary aim of this study was to describe the proportion and characteristics of non-conveyed EMS patients, together with assignment data.

This study showed that approximately 12% of EMS patients in the studied region were not conveyed. This rate is lower than international non-conveyance rates [4, 7]. This difference could relate to structural and contextual differences between EMS systems and how non-conveyance is measured and recorded. For instance, the regional non-conveyance guidelines give EMS clinicians the option to disregard the guidelines if the ambulance nurse feels uncertain about the non-conveyance decision; in this case, the patient may be conveyed even if all other non-conveyance criteria are met.

For adult patients, unspecific symptoms/malaise, abdomen/flank/groin pain and breathing difficulties were the three most commonly used ESS codes documented by the EMS clinician. There are differences between different EMS systems regarding non-conveyed patients’ primary reasons for contacting EMS. For instance, Vloet et al. [16] showed in another context that non-conveyed patients most commonly contacted the EMS due to problems with the circulatory system; injuries; poisoning; and mental, behavioural and neurodevelopmental disorders. To be able to compare and build on previous knowledge, it is important that guidelines and definitions are uniform within and between EMS systems.

In this study, decisions by EMS not to convey patients were not always compatible with the guidelines. Adherence to guidelines in the context of EMS has been shown to be moderate. With adherence to clinical guidelines between 8 and 95%, among which monitoring recommendations are obeyed more frequently than treatment recommendations [36, 37]. The adherence issues could relate to the formats of the guidelines, which make them difficult to use in a prehospital setting [24, 36]. Patient- and organization-related factors such as age, sex, comorbidities, location and education level have also been reported to influence adherence [36].

Patients do not always receive timely treatment due to non-conveyance decisions. Missing data caused by a lack of adherence could cause problems in conducting patient safety evaluations. It is not clear whether adherence issues involving incomplete triage and missing vital signs cause patient safety risks [12, 38, 39]. Adherence to clinical guidelines might improve if measures were taken to clarify for what purpose the guidelines are used and for what purpose medical record data are entered [40]. A digital medical record system with mandatory fields and checkboxes could also potentially mitigate the problem.

To be able to make these complex non-conveyance decisions [11] and follow Swedish legislation [23], ambulance organizations must implement validated guidelines and follow-up systems.

Descriptive studies of the non-conveyance population are beginning to clarify the characteristics of these patients, and further studies are needed to evaluate whether non-conveyance involves patient safety risks, since previous studies have shown contradictory results [25, 26].

For the results to be transferred to other settings, organizational structure, competence, education, transport distances, non-conveyance guidelines, outcome measures, and outcome measure definitions must be taken into consideration.

## Conclusions

Fewer patients were non-conveyed in the studied region compared to national and international non-conveyance rates. There were no clinically relevant differences between sexes in the non-conveyed patient cohort. Some patients with abnormal vital signs and/or missing ESS codes were not conveyed. This study contributes knowledge to the limited research on non-conveyed patients, and it provides information needed to develop validated guidelines that have the potential to enhance the ability of EMS to make patient-safe non-conveyance assessments.

To enhance patient safety, we need further information regarding patients who are not conveyed, in comparison with conveyed patients, in order to understand why they are not conveyed, where they are sent, and whether they experience negative consequences due to this practice. Further research is needed that describes and predicts the outcomes of non-conveyed patients. Predictive models could have the potential to find patients at risk of future deterioration when patients are non-conveyed.

### Limitations

Information bias could distort the proportions of different ESS codes, since not all available codes were used in the studied region. For instance, this limited range of codes could affect the percentage of the ESS code “unspecific symptoms/malaise”, since psychiatric disorders could have been sorted under this code. Since the research data were gathered within the limitations of the organizational structure and triage systems, it was not possible to adjust for this possibility when planning the study. The regional non-conveyance guidelines also give EMS clinicians the option to disregard the guidelines if the ambulance nurse feels uncertain about the non-conveyance decision; in this case, the ambulance may convey the patient even if all other non-conveyance criteria are met. Such departures from the guidelines could have affected the numbers and proportions of non-conveyed patients.

Missing data might also constitute a limitation of the study due to the risk of skewed data and misinterpreted results. Comparative information for conveyed patients was not available for the same period as non-conveyed patients.

## Supplementary information

**Additional file 1.** Exclusion criteria for non-conveyance.

**Additional file 2.** Normal vital signs.

## Data Availability

The datasets generated and analysed during the current study are not publicly available due to patient privacy. The datasets used and analysed during the present study are available from the corresponding author (EH) on reasonable request.

## Abbreviations

ED: Emergency department
EMS: Emergency medical services
ESS: Emergency Signs and Symptoms
RETTS: Rapid Emergency Triage and Treatment System
RN: Registered nurse
