# Supplementary material for: Characteristics of non-conveyed patients in emergency medical services (EMS): a one-year prospective descriptive and comparative study in a region of Sweden
Source: BMC Emerg Med. 2020 Aug 10;20:61. doi: 10.1186/s12873-020-00353-8 (PMC7418316; doi:10.1186/s12873-020-00353-8)
Supplement: Supplementary file 1 — Additional file 1. Exclusion criteria for non-conveyance. [file 12873_2020_353_MOESM1_ESM.pdf]

Cognitive impairment was considered present for the following conditions;

- Drugs/alcohol with clear impact
- Head trauma with signs of concussion
- Severe psychiatric disorder, e.g. psychosis
- Neuropsychiatric functional impairment

Lacking decision-making ability;

- GCS <15, disorientated, cannot account for what has happened or follow instructions.

Is there a history suggestive of acute illness or acute deterioration of chronic disease?

- Affected vital signs >Green, or exclusion criteria >Yellow (>Green for children <18 years)
- Pain VAS >3
- Fever >38,5°C and decreased general condition.
- Suicidal

Required during transport;

- drug administration is needed
- monitoring of vital signs is needed
- medical or nursing care is needed

The ambulance nurse/s feels uncertainty performing the decision to non-convey the patient.
